# Impact of aldehyde dehydrogenase 2 polymorphism on lipid profile alterations in alcohol-abstinent adolescents stratified by steatotic liver disease status

**DOI:** 10.1265/ehpm.26-00078

**Published:** 2026-09-12

**Authors:** Akira Kado, Yukiko Inoue, Kyoji Moriya, Takeya Tsutsumi, Kazuhiko Koike, Mitsuhiro Fujishiro, Shintaro Yanagimoto

**Affiliations:** 1 Department of Gastroenterology, Graduate School of Medicine, The University of Tokyo, 7-3-1 Hongo, Bunkyo-ku, Tokyo 113-8655, Japan; 2 Division for Health Service Promotion, The University of Tokyo, Tokyo, Japan; 3 Division of Infection Control and Prevention, Education Research Center, Tokyo Health Care University, 4-1-17 Higashigotanda, Shinagawa-ku, Tokyo 141-8648, Japan; 4 Department of Infection Control and Prevention, Graduate School of Medicine, The University of Tokyo, Tokyo, Japan; 5 Department of Infectious Diseases, Graduate School of Medicine, The University of Tokyo, Japan; 6 Department of Gastroenterology, Kanto Central Hospital, 6-25-1 Kamiyoga, Setagaya-ku, Tokyo 158-8531, Japan

**Keywords:** Metabolic dysfunction-associated steatotic liver disease, Aldehyde dehydrogenase 2, Lipid profile, Adolescent, Alcohol abstinence, Health checkup

## Abstract

**Background:**

Single-nucleotide polymorphism in aldehyde dehydrogenase 2 (ALDH2) is associated with metabolic dysfunction-associated steatotic liver disease (MASLD). However, this association in alcohol-abstinent individuals remains unknown. We assessed SLD and genotype-inferred ALDH2 activity (ALDH2 activity) in alcohol-abstinent adolescents to explore the association between ALDH2 activity and metabolism-related factors stratified by SLD status.

**Methods:**

In this cross-sectional study, 253 university students (<20 years; 162 males, 91 females) underwent first-year health screening including abdominal ultrasonography and genotyping for ALDH2 rs671, categorized as a high-activity genotype (GG) or reduced-activity genotype (non-GG). The associations of metabolism-related parameters were analyzed across SLD and ALDH2 genotype groups, and predictive performance was assessed using ROC analysis.

**Results:**

We focused on significant changes in metabolism-related parameters, particularly in adolescent males, due to their higher prevalence of SLD. Increased preperitoneal fat and aspartate aminotransferase levels and decreased high-density lipoprotein cholesterol (HDL-C) levels were observed in the non-SLD group, with high ALDH2 activity; low-density lipoprotein cholesterol (LDL-C) levels in the SLD group increased in high ALDH2 activity. In stratified analyses, univariate and multivariate logistic regression revealed that, among adolescents without SLD, those with the GG genotype had significantly lower HDL-C levels (odds ratio [OR], 0.95; 95% confidence interval [CI], 0.91–0.99, p = 0.016). Conversely, among adolescents with SLD, the high-activity GG genotype was associated with significantly higher LDL-C levels (OR, 1.03; 95% CI, 1.01–1.05, p = 0.001). Exploratory cutoffs for HDL-C (<64 mg/dL) and LDL-C (≥114 mg/dL) showed moderate discriminatory ability (AUC 0.727 and 0.701, respectively) and showed modest genotype-classification performance in an independent cohort.

**Conclusions:**

The association between ALDH2 rs671 genotype and lipid profiles differed according to SLD status under non-alcoholic conditions. Combined assessment of SLD and lipid changes may provide an exploratory approach for estimating ALDH2 genotype-inferred alcohol-metabolizing capacity in adolescents.

**Supplementary information:**

The online version contains supplementary material available at https://doi.org/10.1265/ehpm.26-00078.

## Graphical Abstract

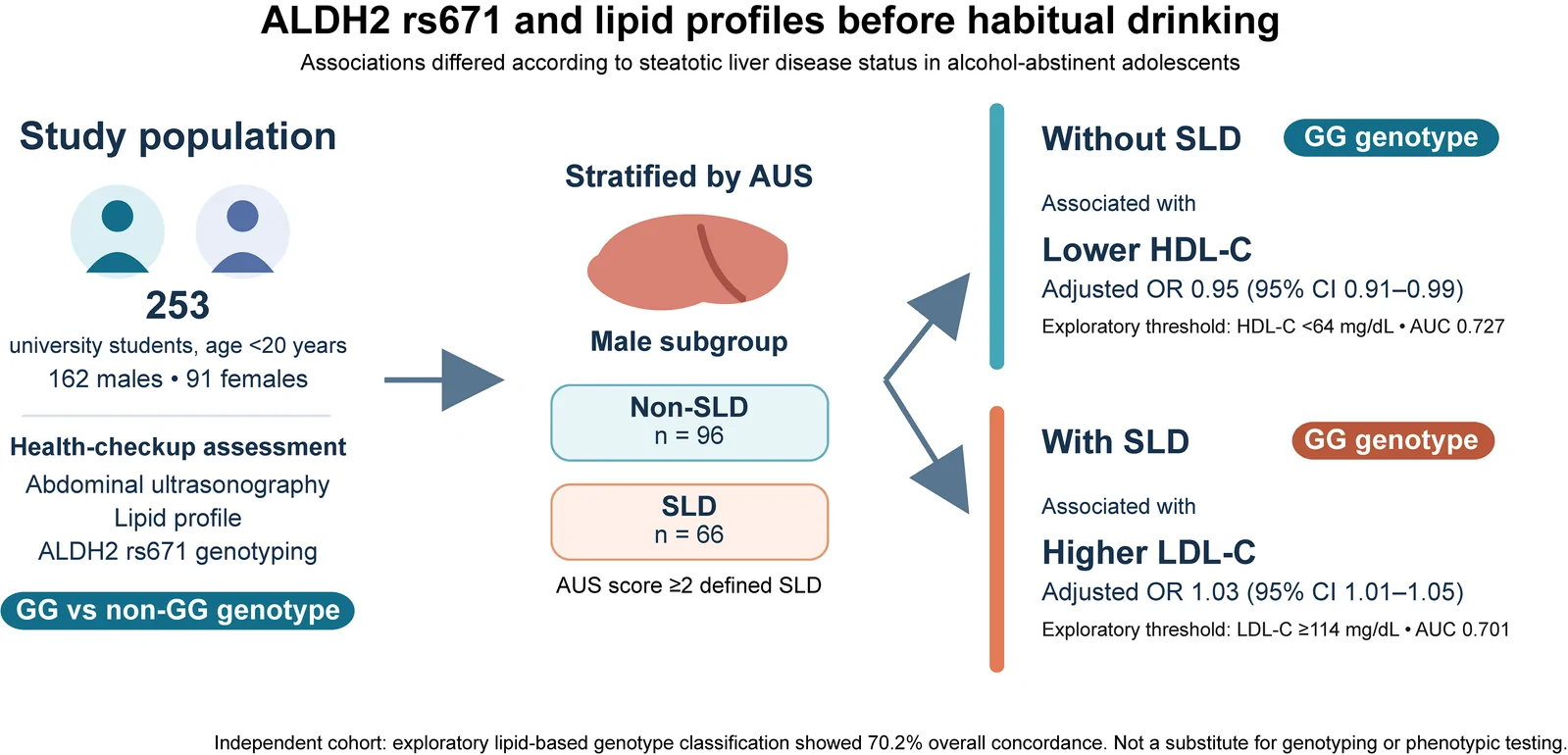

## Background

Metabolic dysfunction-associated steatotic liver disease (MASLD) is the most common chronic liver disease in children and adults worldwide. If not treated, MASLD leads to liver-related events (*e.g.*, liver cirrhosis and cancer) and lifestyle-related diseases, including hypertension (HT), dyslipidemia (DL), diabetes mellitus (DM), and cardiovascular diseases [1–4]. Most people with MASLD have obesity and/or lifestyle-related diseases; however, several lean individuals develop MASLD [2, 3]. This underscores the importance of early diagnosis of steatotic liver disease (SLD) to prevent progression.

SLD is associated with various metabolism-related genetic backgrounds, as a risk allele of the patatin-like phospholipase domain-containing 3 (PNPLA3) single-nucleotide polymorphism (SNP) is associated with non-alcoholic fatty liver disease (NAFLD)/MASLD [5–8]. The PNPLA3 SNP (rs738409) primarily contributes to hepatic fat accumulation by impairing triglyceride hydrolysis in hepatocytes. In contrast, the aldehyde dehydrogenase 2 (ALDH2) SNP (rs671) affects hepatic and systemic metabolism by regulating aldehyde detoxification and modulation of oxidative stress (OS) [9, 10]. Thus, genotype-inferred ALDH2 activity (hereafter, ALDH2 activity) may influence lipid profiles and liver health through distinct mechanisms compared to PNPLA3, particularly in the absence of alcohol consumption.

The ALDH2 SNP increases the risk of SLD development and is associated with poor prognosis [11, 12], while ALDH2 deficiency induces SLD in alcohol-fed mice [13]. Excessive alcohol consumption elevates the risk of health problems, including metabolic syndrome and OS [14, 15]. ALDH2 is involved in alcohol metabolism, aldehyde detoxification, and antioxidant systems [10, 11]. Low ALDH2 activity (GA/AA genotype) increases the risk of alcohol-flushing syndrome and esophageal cancer owing to impaired alcohol metabolism [14, 16]. High activity (GG genotype) has been associated with favorable metabolic profiles in some studies, although the findings have not been consistent [10, 11, 17]. However, in adults, alcohol may be involved in the onset of SLD even if its effect is small and it is properly detoxified by ALDH2. Furthermore, the association between ALDH2 metabolism-related diseases and SLD has been examined in adults with alcohol drinking habits, even if alcohol consumption is low. Nevertheless, this detailed association under strictly non-alcoholic conditions remains unclear.

This study aimed (i) to evaluate SLD and ALDH2 rs671 genotype in alcohol-abstinent adolescents who underwent a first-year university health checkup, (ii) to examine the association between ALDH2 genotype and metabolism-related factors stratified by SLD status, and (iii) to explore the performance of lipid-based thresholds for classifying genotype-inferred ALDH2 activity.

## Methods

### First-year university students

**Fig. 1:**
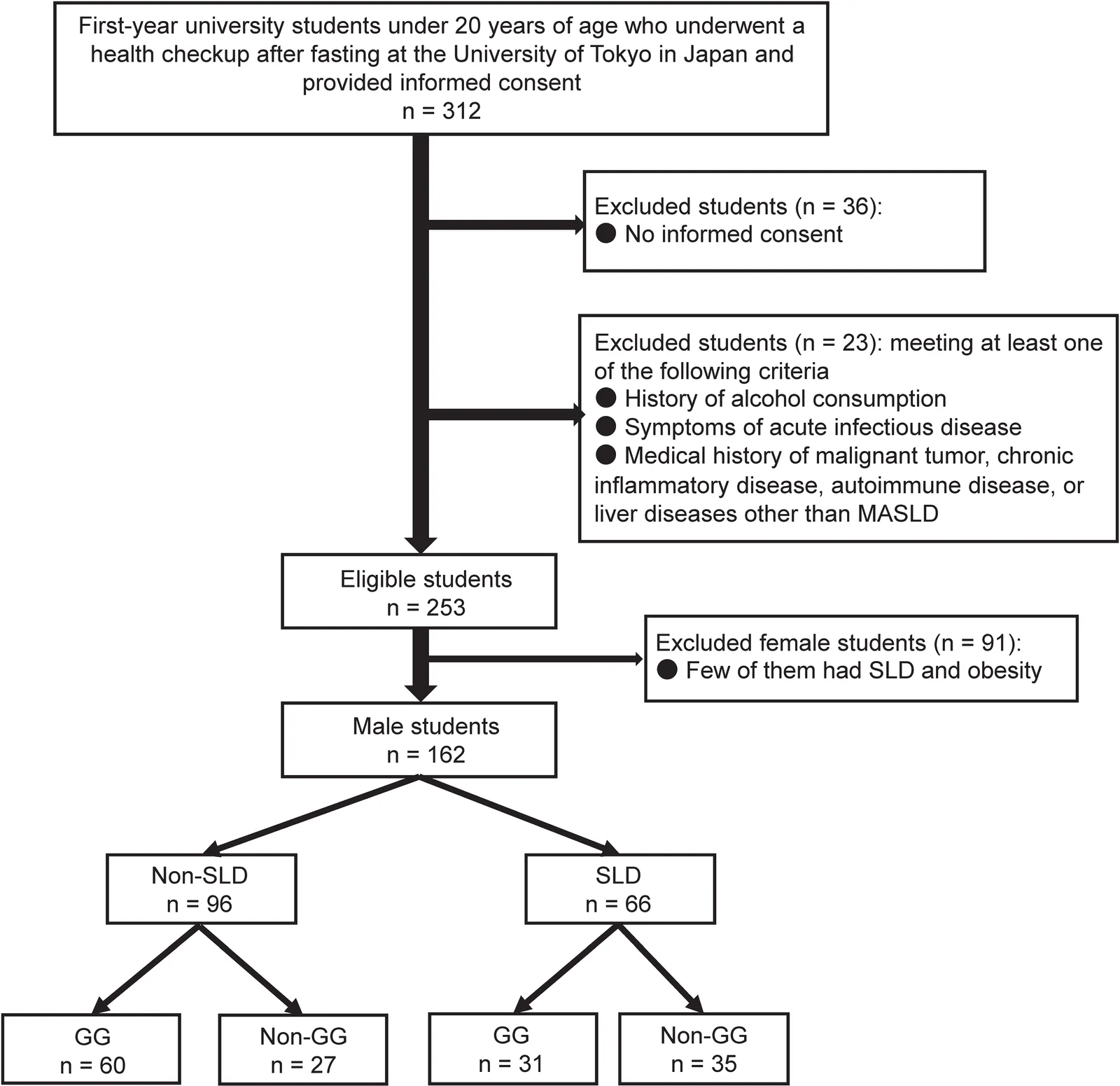
Study flow chart. This study included 253 first-year university students (male:female = 162:91) aged <20 years who underwent health checkups (after an overnight fast) at the University of Tokyo, Japan. Of these male participants, 96 and 66 had non-SLD and SLD, respectively. Of patients with non-SLD and SLD, 60 out of 96 and 31 out of 66 had the ALDH2 GG genotype, respectively.

We recruited a total of 312 Japanese students under 20 years who underwent health checkups at their university in Japan and received informed consent for study participation (Fig. 1). Abdominal ultrasonography (AUS) was conducted as part of the comprehensive health checkup and research protocol among students who consented to participate. Students were included in the present analysis only when complete data on AUS, blood tests, questionnaire-based lifestyle information, and ALDH2 genotyping were available. According to Erikson’s psychosocial theory, these first-year students (18–19 years) were classified as “adolescents,” as previously described [3]. In Japan, participants were considered alcohol-abstinent because Japanese law forbids alcohol use before 20 years. Participants were excluded if they declined consent or had a history of alcohol consumption, acute infection, malignancy, chronic inflammation, autoimmune disease, or liver disease other than MASLD. Overall, eligibility screening led to exclusion of 59 participants who did not meet the criteria, and 253 participants (162 males and 91 females) were enrolled in this study. SLD status was determined using AUS. Obesity was also assessed for comparison with SLD. Obesity was defined as body mass index (BMI) threshold of 25 kg/m^2^, as previously described [3]. Participants were stratified by SLD and obesity status to assess changes in metabolism-related clinical parameters, including waist circumference (WC), systolic and diastolic blood pressure (SBP/DBP), triglycerides (TG), high-density lipoprotein cholesterol (HDL-C), and fasting blood glucose (FBG) [18].

The protocol was approved by the University of Tokyo Clinical Research Review Board (No. 18-277) and adhered to the Declaration of Helsinki. For validation purposes, an independent cohort of 114 male adolescents under 20 years was recruited from the same university during a subsequent health checkup period. The inclusion and exclusion criteria, data collection procedures (including AUS and blood sampling), and genotyping methods were identical to those used in the main cohort. Baseline characteristics were shown in Table S1. Participants provided informed consent and were confirmed to be alcohol-abstinent. This cohort was used to assess the reproducibility of the associations observed in the main analysis, as previously described [3].

### Clinical metabolic parameters

Participants underwent a standard questionnaire, physical examination, and laboratory data. All participants were confirmed to be non-smokers and alcohol-abstinent. Patient age, comorbidities, and medications were recorded. Participants with a history of lifestyle-related diseases and pharmacological treatment for DL, *e.g.* statins or fibrates, were excluded to minimize bias on lipid profiles. The BMI, WC, SBP, and DBP were measured on the day of the health checkup. WC was taken in an upright position at the minimal circumference between the rib cage and iliac crest using a flexible tape. Body weight (BW) and height were measured in light clothing and without footwear [3]. Exercise status was assessed using a brief health-checkup questionnaire, as previously described [3], and was defined as exercise ≥150 min/week. Laboratory analyses covered levels of aspartate aminotransferase (AST), alanine aminotransferase (ALT), γ-glutamyl transpeptidase (GGT), TG, low-density lipoprotein cholesterol (LDL-C), HDL-C, uric acid (UA), creatinine, and C-reactive protein (CRP). FBG and platelet counts were also measured. These metabolic parameters are associated with obesity and SLD [3].

### SLD assessment

SLD was identified using a standardized ultrasonographic scoring protocol, which is commonly applied in adolescent population studies [3, 19]. AUS remains a practical and widely accepted method for large-scale adolescent screening [3, 5]. Controlled attenuation parameter (CAP) and liver stiffness measurement using transient elastography are recognized as valuable tools for quantifying hepatic fat and fibrosis [20]; however, these modalities were not available during the routine health checkups due to time constraints and equipment limitations. Briefly, in the AUS scoring system, AUS findings such as hepatorenal contrast, liver brightness, posterior beam attenuation, and vessel blurring were assessed. To evaluate hepatorenal contrast, long-axis images of the right hepatic lobe and kidney were obtained. Additional paired views of the liver and spleen were captured to compare parenchymal brightness. Scoring was as follows: 1 point for either hepatorenal contrast or increased brightness, 2 points when both were present, and 3 points for marked brightness with contrast. Extra points (1–3) were added for posterior beam attenuation and vessel blurring. Scores were summed, with 0 assigned when neither feature was present. A cumulative score of ≥2 was considered diagnostic for SLD, consistent with histology-based validation. Although categorical ultrasonographic severity grading (mild, moderate, or severe) was not recorded in the health checkup dataset, AUS score was used as an ordinal surrogate marker of ultrasonographic steatosis severity in additional analyses. Two experienced hepatologists independently interpreted the stored images without access to clinical data and resolved discrepancies by consensus to ensure consistency.

Subcutaneous and preperitoneal fat layers were assessed sonographically as surrogates for visceral adiposity. Although computed tomography is a standard method, it is unsuitable for large-scale student screening [3]; therefore, ultrasonography was employed. Preperitoneal fat thickness on AUS served as a practical indicator of visceral fat [21]. Fat thickness was measured at the mid-abdomen using longitudinal AUS.

### Analysis of genetic polymorphisms

All participants were Japanese university students recruited from the same institution. Whole blood samples were acquired from all 367 participants, and genomic DNA was obtained from the peripheral blood using the QIAamp DNA Blood Kit and related reagents (Qiagen, Hilden, Germany) according to the manufacturer’s instructions [15, 22, 23]. The rs671 (ALDH2 family; ALDH2; *n* = 367) was performed through TaqMan allelic discrimination assay (Thermo Fisher Scientific, Waltham, MA, USA) using the appropriate TaqMan primers and probes (Thermo Fisher Scientific; C__11703892_10). Participants were categorized into GG and non-GG (GA/AA) genotype groups; these groups were interpreted as having genotype-inferred high and reduced ALDH2 activity, respectively [5].

### Statistical analysis

All analyses were conducted in JMP 17 software (SAS Institute Japan, Tokyo, Japan). Two-sided p-values below 0.05 were interpreted as statistically significant. The sample size required for this study was calculated using G*Power version 3.1.9.7 (Heinrich-Heine-Universität, Düsseldorf, Germany). Nonparametric variables were compared using the Wilcoxon Kruskal–Wallis test. Data are presented as medians and interquartile ranges (lower to upper quartiles), unless stated otherwise. There were no missing data for the variables included in the analyses. Participants were stratified by SLD and obesity status to examine whether the association between ALDH2 genotype and lipid parameters differed across these subgroups. In additional analyses, exercise status, defined as ≥150 min/week, was examined as a potential lifestyle-related confounder. Logistic regression models were further adjusted for exercise status to assess whether the associations of ALDH2 genotype with HDL-C or LDL-C were independent of exercise status. Univariate and multivariable logistic regression explored associations between ALDH2 genotype and metabolic variables (p < 0.1). False discovery rate correction was applied for multiple comparisons. Models were selected using the Akaike information criterion. Spearman’s rank correlation was used to examine associations between AUS score and adiposity-related parameters, including subcutaneous and preperitoneal fat thickness, BMI, and WC.

## Results

### Participant characteristics

In this study, 253 adolescent students (162 males and 91 females) under 20 years provided written informed consent (Fig. 1). Table 1 summarizes the clinical characteristics of the study participants, including SLD status and ALDH2 SNP. No significant differences in age or ALDH2 SNP were observed between male and female adolescents. SLD was significantly less common in the females than in males. BMI, WC, abdominal fat (preperitoneal and subcutaneous fat), SBP, levels of AST, ALT, GGT, TG, LDL-C, UA, creatinine, and platelet count were significantly higher in the males than in the females, whereas HDL-C levels were significantly lower in the males. Significant sex differences were observed in multiple clinical characteristics, and the prevalence of obesity and SLD was markedly lower in females than in males.

**Table 1:** Clinical and demographic characteristics of all 253 students

**parameter**	**Total** **(n = 253)**	**Male** **(n = 162)**	**Female** **(n = 91)**	**p value**
**Age (years)**	**18 (18–18)**	**18 (18–19)**	**18 (18–18)**	**0.643**
**BMI (kg/m2)**	**21.4 (18.9–24.8)**	**23.8 (20.2–25.9)**	**19.4 (18.3–21.0)**	**<0.001**
**Obese (%)**	**58 (22.9%)**	**57 (35.2%)**	**1 (1.1%)**	
**Non-obese (%)**	**195 (77.1%)**	**105 (64.8%)**	**90 (98.9%)**	
**WC (cm)**	**75 (68.0–85.5)**	**82.3 (72.7–89.0)**	**68.8 (66.4–73.5)**	**<0.001**
**Subcutaneous fat (mm)**	**4.2 (2.1–6.6)**	**5.8 (3.9–7.5)**	**2.1 (1.4–3.3)**	**<0.001**
**Preperitoneal fat (mm)**	**2.9 (1.7–5.2)**	**4.3 (2.4–7.0)**	**1.9 (1.3–2.5)**	**<0.001**
**SBP (mmHg)**	**121 (112–133)**	**127 (114.3–135.0)**	**117 (107.5–122.5)**	**<0.001**
**DBP (mmHg)**	**71 (64–76)**	**71 (64–77)**	**70 (64.5–75.5)**	**0.448**
**AST (U/L)**	**22 (18–26)**	**24 (20.0–28.8)**	**19 (17–22)**	**<0.001**
**ALT (U/L)**	**18 (14–31)**	**25.5 (16.3–40.0)**	**14 (11.0–16.5)**	**<0.001**
**GGT (U/L)**	**19 (15–26)**	**22.5 (17.0–30.8)**	**15 (12.5–17.5)**	**<0.001**
**TG (mg/dL)**	**47 (38–62)**	**51 (39.0–69.8)**	**43 (36.0–51.5)**	**0.002**
**LDL-C (mg/dL)**	**99 (84–114)**	**100.5 (84.0–121.8)**	**94 (81.5–107.0)**	**0.010**
**HDL-C (mg/dL)**	**62 (53–72)**	**56 (50–66)**	**70 (63–81)**	**<0.001**
**UA (mg/dL)**	**5.3 (4.4–6.2)**	**5.9 (5.3–6.6)**	**4.2 (3.8–4.7)**	**<0.001**
**Creatinine (mg/dL)**	**0.75 (0.65–0.84)**	**0.81 (0.75–0.87)**	**0.61 (0.56–0.68)**	**<0.001**
**CRP (mg/dL)**	**0.02 (0.01–0.06)**	**0.03 (0.01–0.07)**	**0.02 (0.01–0.04)**	**0.055**
**FBG (mg/dL)**	**82 (78–86)**	**82 (77.0–85.8)**	**82 (79–87)**	**0.258**
**Platelet count (10,000/µl)**	**27.5 (24.0–31.0)**	**28.5 (25.1–31.5)**	**26.2 (22.1–29.4)**	**<0.001**
**SLD (%)**	**68 (26.9%)**	**66 (40.7%)**	**2 (2.2%)**	**<0.001**
**ALDH2 genotype**				
**GG (%)**	**150 (59.3%)**	**91 (56.2%)**	**59 (64.8%)**	**0.180**
**Non-GG (%)**	**103 (40.7%)**	**71 (43.8%)**	**32 (35.2%)**	
**GA**	**93**	**65**	**28**	
**AA**	**10**	**6**	**4**	

Values for continuous variables were presented as medians (interquartile ranges [Q1, Q3]).

The bold means the parameter significantly differs between males and females.

### Characteristics of male participants with obesity and SLD

Sex-stratified analysis was conducted due to low prevalence of SLD and obesity in females, limiting statistical power for subgroup comparisons. We focused on adolescent males to ensure adequate sample size and account for physiological metabolic differences. The 162 male students were categorized according to two classifications: non-obese and obese groups; non-SLD (score <2) and SLD (score ≥2) groups, based on their AUS scores (Table 2). No significant differences in age or creatinine levels were observed between the two groups. The levels of the remaining clinical parameters, except for HDL-C, were significantly higher in the obese and SLD groups than in the non-obese and non-SLD groups, respectively, whereas HDL-C levels were significantly lower in the obese and SLD groups. Notably, ALDH2 GG genotype was less prevalent in the SLD group, though not significantly. Similar metabolic patterns were observed across the obesity-based and SLD-based classifications.

**Table 2:** Clinical and demographic characteristics in males with obesity and SLD status

**parameter**	**Non-obese** **(n = 105)**	**Obese** **(n = 57)**	**p value**	**Non-SLD** **(n = 96)**	**SLD** **(n = 66)**	**p value**
**Age (years)**	**18 (18–19)**	**18 (18–19)**	**0.180**	**18 (18–19)**	**18 (18–19)**	**0.055**
**BMI (kg/m2)**	**21.9 (18.9–23.8)**	**26.7 (25.8–28.0)**	**<0.001**	**21.3 (18.9–24.2)**	**25.9 (23.9–27.4)**	**<0.001**
**Obese (%)**				**16 (16.7%)**	**41 (62.1%)**	
**Non-obese (%)**				**80 (83.3%)**	**25 (37.9%)**	
**WC (cm)**	**76.5 (69–83)**	**89.7 (86.5–93.5)**	**<0.001**	**76.5 (69–84)**	**89.2 (83.6–93.5)**	**<0.001**
**Subcutaneous fat (mm)**	**4.7 (3.1–6.3)**	**7.5 (5.9–10.7)**	**<0.001**	**4.9 (3.0–6.4)**	**7.2 (5.2–9.4)**	**<0.001**
**Preperitoneal fat (mm)**	**3.2 (2.1–5.1)**	**7.1 (4.1–9.7)**	**<0.001**	**3.2 (1.9–5.1)**	**6.0 (4.1–9.3)**	**<0.001**
**SBP (mmHg)**	**119 (111–131)**	**133 (128–139)**	**<0.001**	**121 (111.0–132.3)**	**131 (121.3–138.0)**	**<0.001**
**DBP (mmHg)**	**68 (62–75)**	**75 (68–82)**	**<0.001**	**68.5 (62.8–75.0)**	**74 (68–82)**	**<0.001**
**AST (U/L)**	**23 (19–26)**	**26 (22–35)**	**0.005**	**22 (18–25)**	**27 (22.0–35.8)**	**<0.001**
**ALT (U/L)**	**20 (16–31)**	**36 (26–52)**	**<0.001**	**19 (14–29)**	**39 (26.5–60.8)**	**<0.001**
**GGT (U/L)**	**20 (17–26)**	**26 (20–36)**	**0.001**	**19 (16.0–24.3)**	**28.5 (21.3–39.0)**	**<0.001**
**TG (mg/dL)**	**47 (37–59)**	**63 (43–91)**	**<0.001**	**45 (36.8–55.0)**	**65.5 (48.0–91.8)**	**<0.001**
**LDL-C (mg/dL)**	**97 (81–111)**	**111 (98–136)**	**<0.001**	**97 (84.0–111.3)**	**111 (92.5–135.3)**	**0.002**
**HDL-C (mg/dL)**	**60 (53–68)**	**52 (46–58)**	**<0.001**	**60 (51.0–69.3)**	**53.5 (47.3–59.0)**	**<0.001**
**UA (mg/dL)**	**5.7 (5.2–6.2)**	**6.4 (5.7–7.1)**	**<0.001**	**5.7 (5.2–6.2)**	**6.2 (5.7–7.2)**	**<0.001**
**Creatinine (mg/dL)**	**0.8 (0.75–0.87)**	**0.82 (0.76–0.86)**	**0.564**	**0.82 (0.76–0.87)**	**0.81 (0.73–0.86)**	**0.281**
**CRP (mg/dL)**	**0.02 (0.01–0.05)**	**0.05 (0.02–0.12)**	**<0.001**	**0.02 (0.01–0.05)**	**0.05 (0.02–0.10)**	**<0.001**
**FBG (mg/dL)**	**80 (76–84)**	**83 (81–88)**	**<0.001**	**80 (76–84)**	**83 (81–88)**	**<0.001**
**Platelet count (10,000/µl)**	**27.4 (24.8–31.2)**	**30 (27.2–32.4)**	**0.026**	**27.0 (23.8–31.2)**	**29.9 (27.2–32.2)**	**0.004**
**SLD (%)**	**25 (23.8%)**	**41 (71.9%)**	**<0.001**			
**ALDH2 genotype**						
**GG (%)**	**61 (58.1%)**	**30 (52.6%)**	**0.506**	**60 (62.5%)**	**31 (47.0%)**	**0.051**
**Non-GG (%)**	**44 (41.9%)**	**27 (47.4%)**		**36 (37.5%)**	**35 (53.0%)**	
**GA**	**39**	**26**		**33**	**32**	
**AA**	**5**	**1**		**3**	**3**	

Values for continuous variables were presented as medians (interquartile ranges [Q1, Q3]).

Bold font indicates that the parameter significantly changed between the non-obese and obese groups and between the non-SLD and SLD groups in males.

### Characteristics of male participants according to ALDH2 SNP

Regarding the ALDH2 SNP, Table 3 presents the clinical characteristics of male adolescents with the GG and non-GG genotypes. No significant differences in age or these metabolic parameters were observed between the two groups, and the prevalence of SLD was lower in the GG genotype group, although not significantly as shown in Table 2. Among adolescent females, no significant differences in these clinical parameters, including the ALDH2 SNP, were observed between the two groups (Table S2). However, among all adolescents, SLD prevalence was significantly lower in the GG genotype group than in the non-GG genotype group in the main cohort (Table S3).

**Table 3:** Clinical and demographic characteristics of ALDH2 GG and non-GG genotypes in males

**parameter**	**GG** **(n = 91)**	**Non-GG** **(n = 71)**	**p value**
**Age (years)**	**18 (18–19)**	**18 (18–19)**	**0.859**
**BMI (kg/m2)**	**23.7 (20.2–25.7)**	**23.9 (20.8–26.1)**	**0.688**
**Obese (%)**	**30 (33.0%)**	**27 (38.0%)**	
**Non-obese (%)**	**61 (67.0%)**	**44 (62.0%)**	
**WC (cm)**	**82.5 (71.5–89.0)**	**82 (74.2–89.3)**	**0.903**
**Subcutaneous fat (mm)**	**5.9 (3.9–7.5)**	**5.1 (3.7–7.5)**	**0.532**
**Preperitoneal fat (mm)**	**4.6 (2.4–7.5)**	**3.8 (2.4–6.4)**	**0.318**
**SBP (mmHg)**	**126 (113.5–133.0)**	**127 (115.5–137.5)**	**0.350**
**DBP (mmHg)**	**70 (63–76)**	**74 (64–78)**	**0.131**
**AST (U/L)**	**24 (21–27)**	**23 (19–32)**	**0.676**
**ALT (U/L)**	**27 (18.0–39.5)**	**23 (16.0–41.5)**	**0.861**
**GGT (U/L)**	**22 (17.5–29.5)**	**23 (17–31)**	**0.829**
**TG (mg/dL)**	**49 (39–69)**	**55 (39.0–70.5)**	**0.340**
**LDL-C (mg/dL)**	**104 (84–122)**	**100 (88–113)**	**0.596**
**HDL-C (mg/dL)**	**55 (49.5–63.5)**	**58 (50.5–68.0)**	**0.145**
**UA (mg/dL)**	**5.9 (5.4–6.6)**	**5.9 (5.3–6.7)**	**0.962**
**Creatinine (mg/dL)**	**0.82 (0.76–0.86)**	**0.8 (0.74–0.87)**	**0.341**
**CRP (mg/dL)**	**0.03 (0.01–0.07)**	**0.03 (0.01–0.07)**	**0.866**
**FBG (mg/dL)**	**81 (77.5–85.0)**	**83 (76–87)**	**0.340**
**Platelet count (10,000/µl)**	**28.4 (25.1–31.1)**	**28.6 (25.1–32.2)**	**0.611**
**SLD (%)**	**31 (34.1%)**	**35 (49.3%)**	**0.051**

Values for continuous variables were presented as medians (interquartile ranges [Q1, Q3]).

Next, the aforementioned metabolic parameters were examined between the ALDH2 GG and non-GG genotypes in adolescent males with non-SLD and SLD, as the males had different metabolic patterns (Table 4). In the non-SLD group, preperitoneal fat and AST levels were significantly higher in the GG genotype group than in the non-GG genotype group, whereas HDL-C levels were significantly lower in the GG genotype group. In the SLD group, only LDL-C levels were significantly higher in the GG genotype group. The same analyses were performed in male adolescents with and without obesity (Table S4). In the non-obese group, only HDL-C levels were significantly lower in the GG genotype than in the non-GG genotype. In the obese group, only LDL-C levels were significantly higher in the GG genotype.

**Table 4:** Clinical and demographic characteristics in males with high ALDH2 activity according to SLD status

**parameter**	**Non-SLD**			**SLD**		

	**GG** **(n = 60)**	**Non-GG** **(n = 36)**	**p value**	**GG** **(n = 31)**	**Non-GG** **(n = 35)**	**p value**
Age (years)	18 (18–18)	18 (18–18)	0.954	18 (18–19)	18 (18–19)	0.680
BMI (kg/m2)	23.0 (19.4–24.2)	21.0 (18.6–24.0)	0.261	25.9 (24.4–27.9)	25.9 (24.0–27.2)	0.827
Obese (%)	10 (16.7%)	6 (16.7%)		20 (64.5%)	21 (60.0%)	
Non-obese (%)	50 (83.3%)	30 (83.3%)		11 (35.5%)	14 (40.0%)	
WC (cm)	78.2 (70.5–84.7)	74.6 (66.2–81.3)	0.066	89 (84.8–94.5)	89.5 (82.8–92.8)	0.758
Subcutaneous fat (mm)	5.4 (3.0–6.7)	4.6 (3.1–5.7)	0.183	6.9 (5.8–10.0)	7.4 (4.9–9.0)	0.546
**Preperitoneal fat (mm)**	**3.4 (2.2–5.4)**	**2.5 (1.6–4.0)**	**0.039**	6.4 (4.3–9.5)	5.6 (3.8–8.9)	0.492
SBP (mmHg)	120.5 (112–132)	121.5 (109.5–133.0)	0.847	131 (124.5–136.0)	133 (121.0–141.5)	0.355
DBP (mmHg)	67.5 (62–75)	69 (63.0–75.3)	0.462	72 (67.5–82.5)	75 (68.0–81.5)	0.537
**AST (U/L)**	**23 (20–26)**	**20.5 (17.0–23.3)**	**0.029**	25 (22.5–33.0)	30 (22–37)	0.403
ALT (U/L)	21 (16–29)	17 (14–21)	0.061	39 (25.0–51.5)	39 (28.0–72.5)	0.516
GGT (U/L)	19 (16.8–23.0)	19.5 (15.0–26.3)	0.982	31 (24.5–40.5)	26 (20.5–38.0)	0.167
TG (mg/dL)	45 (37.0–54.3)	45 (35.8–57.5)	0.788	69 (50.5–103.5)	62 (49–85)	0.567
**LDL-C (mg/dL)**	97.5 (83.3–112.0)	96 (86.3–110.3)	0.961	**121 (100.0–144.5)**	**105 (84.0–117.5)**	**0.011**
**HDL-C (mg/dL)**	**56.5 (50.0–64.3)**	**67 (56.8–75.0)**	**0.001**	54 (49.5–61)	53 (47–58)	0.483
UA (mg/dL)	5.8 (5.3–6.3)	5.6 (5.1–6.0)	0.103	6.2 (5.5–7.05)	6.2 (5.8–7.15)	0.286
Creatinine (mg/dL)	0.83 (0.78–0.86)	0.79 (0.76–0.87)	0.405	0.81 (0.73–0.85)	0.8 (0.73–0.85)	0.758
CRP (mg/dL)	0.02 (0.01–0.06)	0.02 (0.01–0.04)	0.793	0.06 (0.02–0.10)	0.04 (0.03–0.09)	0.628
FBG (mg/dL)	81 (77–84)	78.5 (75.0–85.3)	0.476	83 (79.5–88.0)	84 (82–88)	0.341
Platelet count (10,000/µl)	26.9 (24.9–31.2)	27.4 (22.6–31.5)	0.625	29.7 (27.4–30.6)	30.2 (27.2–32.6)	0.426

Values for continuous variables were presented as medians (interquartile ranges [Q1, Q3]).

Bold font indicates that the parameter significantly changed between the GG and non-GG genotypes in the non-SLD and SLD groups in males.

### Indicators of the high-activity ALDH2 genotype in male participants

We used logistic regression to identify metabolic parameters associated with the high-activity GG genotype in male adolescents stratified by SLD status (Table 5). In univariate analysis, WC, preperitoneal fat, and levels of AST and HDL-C were significantly associated with the GG genotype in the non-SLD group, and only the LDL-C level was significant in the SLD group. In multivariable analysis, HDL-C and LDL-C were independently associated with the GG genotype in the non-SLD and SLD groups, respectively. Identification of metabolic indicators of the GG genotype in non-obese and obese male adolescents was also examined; no significant independent indicator of the GG genotype was observed in the non-obese group, whereas LDL-C level was a significant independent indicator in the obese group (Table S5).

**Table 5:** Metabolic parameters associated with the GG genotype according to SLD status in male participants

**parameter**	**Non-SLD (n = 96)**						**SLD (n = 66)**					

	**Univariate**			**Mutivariate**			**Univariate**			**Mutivariate**		

	**Odds Ratio**	**95% CI**	***p***	**Odds Ratio**	**95% CI**	***p***	**Odds Ratio**	**95% CI**	***p***	**Odds Ratio**	**95% CI**	***p***
WC (cm)	1.05	0.99–1.10	0.074	1.00	0.94–1.07	0.883	1.02	0.96–1.07	0.562			
Preperitoneal fat (mm)	1.26	1.02–1.54	0.018	1.13	0.91–1.45	0.269	1.04	0.89–1.21	0.617			
AST (U/L)	1.06	0.99–1.13	0.082	1.04	0.97–1.13	0.26	0.98	0.94–1.02	0.179			
ALT (U/L)	1.02	0.99–1.06	0.155				0.99	0.97–1.00	0.168			
**LDL-C (mg/dL)**	1.00	0.98–1.01	0.846				**1.02**	**1.01–1.04**	**0.006**	**1.03**	**1.01–1.05**	**0.001**
**HDL-C (mg/dL)**	**0.94**	**0.91–0.98**	**0.002**	**0.95**	**0.91–0.99**	**0.016**	1.02	0.97–1.08	0.403			

Logistic regression analysis was used for univariate and multivariate analyses of the association between significant metabolic parameters and the GG genotype in male participants stratified by SLD status.

As exercise habits may influence lipid profiles, exercise status was additionally examined as a potential lifestyle-related confounder. Exercise status, defined as ≥150 min/week, did not differ significantly according to ALDH2 genotype in male participants, either overall or after stratification by SLD status (Table S6, panel A). In exercise-adjusted logistic regression models, lower HDL-C remained significantly associated with the GG genotype in the non-SLD group, whereas higher LDL-C remained significantly associated with the GG genotype in the SLD group (Table S6, panel B). In addition, AUS score was analyzed as an ordinal surrogate marker of ultrasonographic steatosis severity; AUS score was significantly correlated with adiposity-related parameters, including preperitoneal fat thickness, subcutaneous fat thickness, BMI, and WC, in male participants (Table S6, panel C). Among males with SLD, AUS score was also significantly correlated with preperitoneal fat thickness, BMI, and WC, but not with subcutaneous fat thickness. AUS score did not significantly differ according to ALDH2 genotype among males with SLD.

**Fig. 2:**
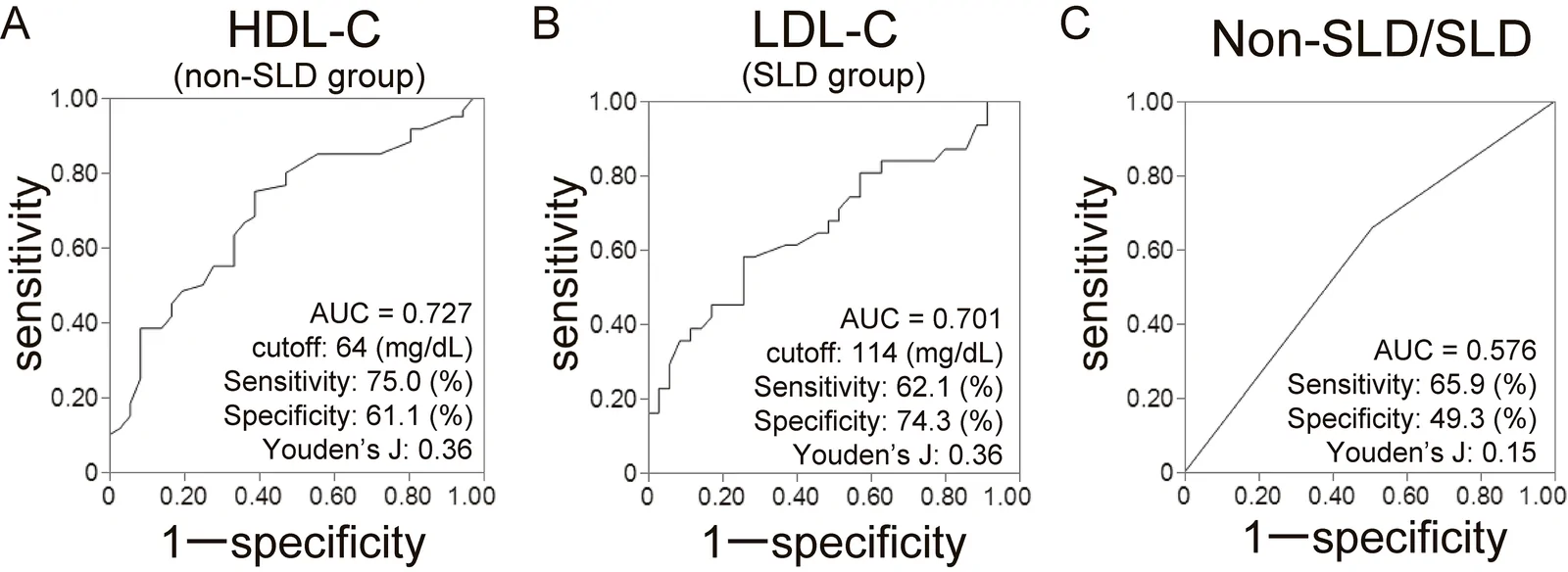
ROC analysis of lipid indicators for classifying the ALDH2 GG genotype in males. AUC values for (A) HDL-C level in the non-SLD group, (B) LDL-C level in the SLD group, and (C) SLD status.

Furthermore, the discriminatory performance of HDL-C and LDL-C for classifying GG versus non-GG genotype in male adolescents with non-SLD and SLD was assessed based on the HDL-C level in the non-SLD group, LDL-C level in the SLD group, and SLD status using the area under the curve (AUC). For comparison, the discriminatory performance of SLD status for classifying GG versus non-GG genotype was also assessed, as shown in Table 2 and Tables S1. The ROC analysis was conducted using threshold values of 64 for HDL-C and 114 for LDL-C in the non-SLD and SLD groups, respectively, using Youden’s index (J) (Fig. 2).

The AUC was 0.727 for HDL-C in the non-SLD group, 0.701 for LDL-C in the SLD group, and 0.576 for SLD status in the overall male cohort. HDL-C in the non-SLD group and LDL-C in the SLD group showed moderate discriminatory performance for classifying GG versus non-GG genotype in adolescent males. These findings identified HDL-C <64 mg/dL in the non-SLD group and LDL-C ≥114 mg/dL in the SLD group as exploratory thresholds for classifying participants according to GG versus non-GG genotype within the corresponding SLD strata.

### Evaluation of exploratory lipid thresholds in an independent male cohort

The exploratory HDL-C and LDL-C thresholds derived in the main cohort were evaluated in an independent cohort of 114 adolescent males. Participants who met the eligibility criteria and provided consent underwent AUS and blood tests to assess SLD, HDL-C, LDL-C, and ALDH2 SNP. Table S1 presents the clinical characteristics of the participants, including SLD status (non-SLD:SLD = 77:37) and ALDH2 SNP and shows the same lipid changes as described in Table 4. Using these exploratory lipid thresholds for genotype classification, 30 of 38 non-SLD participants with HDL-C <64 and 14 of 17 SLD participants with LDL-C ≥114 had the GG genotype, and 25 of 39 non-SLD participants with HDL-C ≥64 and 11 of 20 SLD participants with LDL-C <114 had the non-GG genotype (Table 6). The lipid-based classification was concordant with the observed ALDH2 genotype in 80 of 114 participants (70.2%). These exploratory thresholds showed modest performance for classifying GG versus non-GG genotype. In this validation cohort, the association between the non-GG genotype and SLD status observed in the main overall cohort was not replicated. SLD was observed in 23 of 67 participants with the GG genotype and 14 of 47 participants with the non-GG genotype, and this difference was not statistically significant.

**Table 6:** Performance of exploratory lipid thresholds for classifying ALDH2 genotype-inferred activity

**AUS**		**Non-SLD** **(n = 77)**		**SLD** **(n = 37)**	
Lipid changes		HDL-C < 64	HDL-C ≥ 64	LDL-C ≥ 114	LDL-C < 114
Classification based on the exploratory lipid threshold		GG	Non-GG	GG	Non-GG
		38	39	17	20
Observed ALDH2 genotype	GG	**30**	14	**14**	9
	Non-GG	8	**25**	3	**11**
Overall classification accuracy		Overall concordance: **70.2**% (80/114)			

## Discussion

In this study, we examined the associations of ALDH2 rs671 genotype with SLD status and metabolism-related factors in alcohol-abstinent adolescents. We found that the association of ALDH2 rs671 genotype with HDL-C and LDL-C differed according to SLD status in alcohol-abstinent adolescents. Specifically, the GG genotype was associated with lower HDL-C in the non-SLD group and higher LDL-C in the SLD group. The exploratory HDL-C and LDL-C thresholds showed modest genotype-classification performance in an independent cohort. These findings suggest that ALDH2 genotype-related lipid patterns may be detectable before habitual alcohol consumption begins, although their clinical utility remains to be established.

GG genotype accounted for 50–60% of ALDH2 SNPs, consistent with previous reports [24]. In adults, low ALDH2 activity increases SLD risk [11, 12, 25], but alcohol consumption complicates interpretation. The relationship between the risk of SLD and low and high ALDH2 metabolic activity in adults without alcohol consumption is unknown. In the main cohort, low ALDH2 activity was associated with a higher prevalence of SLD in the overall adolescent population (Table S3). However, this association was not replicated in the independent validation cohort. Therefore, the potential association between low ALDH2 activity and SLD susceptibility should be interpreted cautiously and requires further validation.

The association between ALDH2 genotype and SLD prevalence should be distinguished from the genotype–lipid associations observed after stratification by SLD status, because these represent different outcomes. In the main cohort, the non-GG genotype was associated with a higher prevalence of SLD in the overall adolescent population, but this association was not reproduced in the independent cohort. In contrast, among males stratified by SLD status, the GG genotype was associated with lower HDL-C in the non-SLD group and higher LDL-C in the SLD group. These findings suggest that SLD status may modify ALDH2 genotype-related lipid patterns rather than indicating that either genotype-inferred high or low ALDH2 activity is uniformly metabolically unfavorable.

ALDH2 SNPs are associated with obesity-related parameters, such as BMI, WC, and preperitoneal fat in adults [26]; ALDH2 activity may also be associated with obesity. However, no significant association was observed between ALDH2 activity and obesity status in adolescent males (Table 2). Unlike adults, obesity may not directly reflect ALDH2-related metabolic phenotypes in adolescents.

Moderate alcohol consumption affects lipid metabolism, lowers LDL-C, and elevates HDL-C; however, the degree of lipid abnormalities varies depending on the amount of alcohol consumption [27–30]. Heavy alcohol consumption is associated with an elevated risk of heart disease, including elevated TG levels and HT risk [28]. Among individuals with high ALDH2 activity, who consume a relatively large amount of alcohol, the levels of total cholesterol and LDL-C tend to increase through inhibition of lipid degradation [31, 32]. Hepatic insulin resistance due to high alcohol intake induces metabolic abnormalities with high ALDH2 activity [33], which may also increase the risk of DM. However, racial and regional differences in drinking habits may have attenuated the association between ALDH2 SNPs and DL. The intrinsic functions of ALDH2 and the alcoholic effects on DL and metabolic syndrome are confounded; therefore, these effects under non-alcoholic conditions should be examined. This study focused exclusively on alcohol-abstinent adolescents and may provide valuable insights into the function of ALDH2 in lipid metabolism, depending on the degree of hepatic steatosis.

ALDH2 is a novel regulator of cholesterol biosynthesis via several signaling pathways and may play an important role in cardiovascular diseases [34–36]. Among several metabolic factors, high ALDH2 activity increases the risk of type 2 DM and is associated with higher BMI, BP, FBG, TG, and LDL-C levels, and HT risk [37, 38]. However, these studies have frequently focused on adults with light-to-heavy alcohol drinking habits. In the current study, no significant differences in these metabolic factors were observed between high and low ALDH2 activity in alcohol-abstinent adolescents (Table S3). ALDH2 metabolic activity alleviates OS and inflammation, promotes β-oxidation, and reduces lipogenesis [31]. Low ALDH2 activity is linked to mortality, especially without DL, due to reduced ROS detoxification [11]. Regarding several metabolic parameters related to lifestyle-related diseases, different findings between high and low ALDH2 activity have been observed depending on the pathological conditions and are controversial, even in lipid metabolism. It remains unclear whether high or low ALDH2 activity aggravates lifestyle-related diseases.

Among metabolic factors, HDL-C and LDL-C have been extensively studied in relation to ALDH2 metabolic activity, especially in adults. Several previous studies have examined ALDH2 rs671 and lipid metabolism under non-drinking or alcohol-adjusted conditions [34]. For example, Wada et al. reported that ALDH2 variation was associated with HDL-C among Japanese non-drinkers, supporting an alcohol-independent role of ALDH2 in HDL regulation. Meta-analytic evidence in Asian populations has also suggested that rs671 influences lipid levels, although the magnitude and direction of these associations may differ by sex, ethnicity, age, and alcohol exposure [38]. Compared with these previous adult studies, this study focused on alcohol-abstinent adolescents before habitual drinking began and further stratified the analyses according to SLD status.

ALDH2 regulates HDL biogenesis via the LXRα–ABCA1 pathway, even in non-drinkers [39]; ALDH2 may affect HDL-C through alcohol-independent mechanisms. ALDH2 also modulates OS and mitochondrial function, influencing HDL turnover [10]. These findings support the plausibility that ALDH2 polymorphism could influence HDL-C levels via intrinsic pathways. ALDH2 SNPs affect HDL-C levels [34, 36], and low ALDH2 activity is negatively associated with HDL-C levels in adult men [40]. In adults with alcohol drinking habits, high ALDH2 activity and heavy alcohol intake tend to increase HDL-C by suppressing HDL degradation [27, 28, 41]. Regarding LDL-C, ALDH2 SNPs may suppress the transcription of lysosomal H+ pump subunits in the nucleus, which are important for lipid degradation and foam cell formation [42]. The interaction between ALDH2 and the LDL receptor may directly act on ATP6V0E2 (a substance important for maintaining lysosomal function and degrading oxidized LDL-C) to increase foam cell formation [42].

In this study, the GG genotype was associated with lower HDL-C levels in the non-SLD group and higher LDL-C levels in the SLD group among alcohol-abstinent adolescents (Table 5). These associations may reflect alcohol-independent differences in HDL and LDL metabolism related to ALDH2 genotype and hepatic lipid accumulation; however, mechanistic studies are required. In contrast, TG levels did not differ significantly between the GG and non-GG genotypes, regardless of SLD status. One possible explanation is that TG levels were generally low in this young health-checkup-based population, limiting the ability to detect genotype-related differences. In addition, the absence of habitual alcohol exposure and the younger age of the participants may have attenuated TG-related metabolic differences that are often observed in adult populations.

This impact of ALDH2 on HDL-C and LDL-C may serve as an exploratory health-checkup-based indicator of ALDH2 genotype-inferred activity in alcohol-abstinent adolescents (Table 6). Importantly, these lipid thresholds should not be interpreted as clinical diagnostic cutoffs for DL or as substitutes for ALDH2 genotyping or established phenotypic tests such as the ethanol patch test [43]. Rather, they may provide adjunctive information for identifying adolescents in whom ALDH2-related alcohol-metabolizing capacity could be considered during preventive counseling.

ALDH2 deficiency (G>A, rs671) is most prevalent in Asian countries, including Japan, and increases cancer risk [44]. Low ALDH2 activity increases the risk of alcohol-flushing syndrome and esophageal cancer [14, 16]. The combination of ALDH2 metabolic activity and alcohol consumption is associated with increased hepatocellular carcinoma and mortality [45]. ALDH2 SNP increased the risk of cardiovascular disease, HT, type 2 DM, and DL [34–38]. Therefore, early awareness of ALDH2-related alcohol-metabolizing capacity during adolescence may be valuable for preventive education before drinking habits develop.

Compared with the ethanol patch test, this lipid-based indicator is indirect and exploratory; however, HDL-C, LDL-C, and AUS findings are already available in many health-checkup settings [3]. Confirmatory ALDH2 genotyping or established phenotypic testing remains necessary when accurate assessment of alcohol-metabolizing capacity is required. Thus, the combination of SLD status and lipid profile changes may provide an exploratory approach for estimating ALDH2 genotype-inferred alcohol-metabolizing capacity in adolescents; however, its clinical utility requires prospective validation before implementation.

This study has several limitations. First, this study obtained data during health check-ups in a single country. Regional and ethnic differences may affect non-SLD and SLD in adolescents; further studies are needed [46]. Second, the cohort’s mean BMI exceeded the national reference for Japanese males aged 15–19 years [2, 3, 47]. As participants were students who underwent comprehensive health assessment including AUS and genotyping, the cohort may not fully represent the general population of Japanese adolescents. This selection may partly explain the relatively high BMI and SLD prevalence among male participants. Third, the extremely low prevalence of obesity and SLD in adolescent females precluded sufficient analysis of ALDH2 metabolic activity. Sex-based physiological differences in Table 1 suggest that female metabolic factors may differ. Future studies should collect data on female adolescents. Fourth, ALDH2 detoxifies acetaldehyde from alcohol; however, acetaldehyde also arises endogenously through ROS, diet, and gut/oral bacteria [15, 48, 49]. Acetaldehyde effects may partially persist even in alcohol-abstinent adolescents and warrant future study. Finally, although ALDH2 metabolic activity may affect lipid profiles, the causality remains unclear due to possible confounders. BMI, WC, visceral fat, and lifestyle habits, *e.g.* dietary intake and physical activity, may influence lipid metabolism and interact with ALDH2 pathways [33]. AUS score was analyzed as an ordinal surrogate marker of ultrasonographic steatosis severity, as quantitative assessment using CAP or MRI-PDFF was unavailable. Although the associations between ALDH2 activity and HDL-C or LDL-C remained significant after adjustment for exercise status, exercise was assessed using a brief health-checkup questionnaire, as previously described [3], rather than a validated detailed physical activity instrument. Therefore, sedentary time, exercise intensity, sports type, daily activity level, and longitudinal lifestyle changes before and after university entrance were not fully captured. Although multivariable and exercise-adjusted analyses were performed, residual lifestyle-related confounding cannot be ruled out. Future mechanistic and longitudinal studies using quantitative liver fat assessment and detailed lifestyle evaluation are crucial to delineate the causal relationship between ALDH2 polymorphism and lipid regulation.

## Conclusions

In alcohol-abstinent adolescent males, the association between ALDH2 rs671 genotype and lipid profiles differed according to SLD status: the GG genotype was associated with lower HDL-C in the non-SLD group and higher LDL-C in the SLD group. These associations remained significant after adjustment for exercise status. Exploratory HDL-C and LDL-C thresholds showed modest concordance with ALDH2 genotype in an independent cohort; however, these thresholds should not be considered alternatives to ALDH2 genotyping or established phenotypic testing. The association between the non-GG genotype and higher SLD prevalence observed in the main cohort was not reproduced in the independent cohort. Further prospective studies are required to clarify the mechanisms and clinical relevance of the associations between ALDH2 genotype and lipid profiles observed after stratification by SLD status.

## Abbreviations

MASLD: metabolic dysfunction-associated steatotic liver disease
HT: hypertension
DL: dyslipidemia
DM: diabetes mellitus
SLD: steatotic liver disease
PNPLA3: patatin-like phospholipase domain-containing 3
SNP: single-nucleotide polymorphism
NAFLD: non-alcoholic fatty liver disease
ALDH2: aldehyde dehydrogenase 2
OS: oxidative stress
AUS: abdominal ultrasonography
BMI: body mass index
SBP: systolic blood pressure
DBP: diastolic blood pressure
WC: waist circumference
TG: triglyceride
HDL: high-density lipoprotein
FBG: fasting blood glucose
BW: body weight
AST: aspartate aminotransferase
ALT: alanine aminotransferase
GGT: γ-glutamyl transpeptidase
LDL: low-density lipoprotein
UA: uric acid
CRP: C-reactive protein
CAP: controlled attenuation parameter
CT: computed tomography
AUC: area under the curve

## Supplementary information

**Additional file 1:** **Table S1** Clinical characteristics in another cohort males with the ALDH2 GG genotype according to SLD status. **Table S2** Clinical and demographic characteristics of ALDH2 GG and non-GG genotype in females. **Table S3** Clinical and demographic characteristics of ALDH2 GG and non-GG genotype in all 253 students. **Table S4** Clinical and demographic characteristics in males with the ALDH2 GG genotype according to obesity status. **Table S5** Metabolic parameters associated with the GG genotype according to obesity status in male participants. **Table S6** Additional analyses of exercise status and ultrasonographic steatosis score in male participants.

**Additional file 2:** Completed STROBE Statement checklist for this cross-sectional study.

## Declarations

### Availability of data and materials

The data of this study are available on request from the corresponding author. The data are not publicly available due to privacy reasons.
