# Supplementary material for: Impact of aldehyde dehydrogenase 2 polymorphism on lipid profile alterations in alcohol-abstinent adolescents stratified by steatotic liver disease status
Source: Environ Health Prev Med. 2026 Sep 12;31:60. doi: 10.1265/ehpm.26-00078 (PMC13585472; doi:10.1265/ehpm.26-00078)
Supplement: Supplementary file 1 [file ehpm-31-060-s001.docx]

**Supplementary file (Additional file 1)**

**Table S1** Clinical characteristics in another cohort males with the ALDH2 GG genotype according to SLD status

Values for continuous variables are median (interquartile range [Q1, Q3]).

The bold indicates the parameter significantly changes between GG and non-GG genotype in non-SLD and SLD group in males.

**Table S2** Clinical and demographic characteristics of ALDH2 GG and non-GG genotype in females

Values for continuous variables are median (interquartile range [Q1, Q3]).

**Table S3** Clinical and demographic characteristics of ALDH2 GG and non-GG genotype in all 253 students

Values for continuous variables are median (interquartile range [Q1, Q3]).

The bold indicates the parameter significantly changes between GG and non-GG genotype in all students.

**Table S4** Clinical and demographic characteristics in males with the ALDH2 GG genotype according to obesity status

Values for continuous variables are median (interquartile range [Q1, Q3]).

The bold indicates the parameter significantly changes between GG and non-GG genotype in non-obese and obese group in males.

**Table S5** Metabolic parameters associated with the GG genotype according to obesity status in male participants

Logistic regression analysis was performed to assess the association between significant metabolic parameters and the GG genotype in male participants stratified by obesity status. Obesity was defined as BMI ≥25 kg/m². Variables with p < 0.1 in univariate analyses were considered for multivariable models. Models were selected using the Akaike information criterion. Bold values indicate statistically significant associations (p < 0.05).

**Table S6** Additional analyses of exercise status and ultrasonographic steatosis score in male participants

Panel A. Exercise status according to ALDH2 genotype

Panel B. Exercise-adjusted logistic regression for the ALDH2 GG genotype

Panel C. Correlations between AUS score and adiposity-related parameters

Panel A shows the distribution of exercise status, defined as ≥150 min/week, according to ALDH2 genotype. Panel B shows exercise-adjusted logistic regression models for the ALDH2 GG genotype in male participants stratified by SLD status. Panel C shows Spearman’s rank correlations between AUS score and adiposity-related parameters. Data in Panel A are shown as n/N (%). Bold values indicate statistically significant group differences, associations, or correlations (p < 0.05).
