# Supplementary material for: Impact of aldehyde dehydrogenase 2 polymorphism on lipid profile alterations in alcohol-abstinent adolescents stratified by steatotic liver disease status
Source: Environ Health Prev Med. 2026 Sep 12;31:60. doi: 10.1265/ehpm.26-00078 (PMC13585472; doi:10.1265/ehpm.26-00078)
Supplement: Supplementary file 2 [file ehpm-31-060-s002.docx]

STROBE Statement—checklist of items that should be included in reports of observational studies

**Impact of aldehyde dehydrogenase 2 polymorphism on lipid profile alterations in alcohol-abstinent adolescents stratified by steatotic liver disease status**

| **Section/item** | **No.** | **STROBE recommendation** | **Page no.** | **Relevant text from manuscript** |
| --- | --- | --- | --- | --- |
| **Title and abstract** | 1a | Indicate the study design with a commonly used term in the title or abstract. | 3–4 | Abstract Methods: “In this cross-sectional study...” |
|  | 1b | Provide an informative and balanced summary of what was done and found. | 3–4 | Structured abstract: Background, Methods, Results, and Conclusions. |
| **Introduction: Background/rationale** | 2 | Explain the scientific background and rationale. | 5–6 | Background: rationale for examining ALDH2 rs671, lipid profiles, and SLD under alcohol-abstinent conditions. |
| **Introduction: Objectives** | 3 | State specific objectives, including any prespecified hypotheses. | 6 | Final paragraph of Background: three study aims. |
| **Methods: Study design** | 4 | Present key elements of study design early in the paper. | 3–4, 6–12 | Abstract Methods; First-year university students; Statistical analysis. |
| **Methods: Setting** | 5 | Describe setting, locations, and relevant periods of recruitment and data collection. | 6–8 | University health-checkup setting in Japan; independent cohort recruited during a subsequent health-checkup period. |
| **Methods: Participants** | 6a | Give eligibility criteria and sources and methods of participant selection for a cross-sectional study. | 6–8, 36, 48 | First-year university students; eligibility, exclusions, consent, recruitment flow, and Figure 1. |
|  | 6b | For matched studies, give matching criteria and numbers. | NA | Not applicable; this was not a matched study. |
| **Methods: Variables** | 7 | Define outcomes, exposures, predictors, potential confounders, effect modifiers, and diagnostic criteria. | 7–11 | Clinical parameters; SLD assessment; ALDH2 rs671 genotype; exercise status; obesity and SLD definitions. |
| **Methods: Data sources/measurement** | 8 | For each variable, give data sources and assessment methods. | 8–11 | Questionnaire, physical examination, laboratory testing, AUS protocol, fat thickness measurement, and TaqMan genotyping. |
| **Methods: Bias** | 9 | Describe efforts to address potential sources of bias. | 6–12, 22–24 | Eligibility restrictions, medication exclusions, blinded AUS interpretation, stratified and adjusted analyses; limitations discussed. |
| **Methods: Study size** | 10 | Explain how the study size was arrived at. | 11 | Statistical analysis: sample size calculation using G*Power 3.1.9.7. |
| **Methods: Quantitative variables** | 11 | Explain how quantitative variables were handled and groupings chosen. | 7, 9–11, 14–16, 47 | BMI and AUS thresholds; nonparametric analyses; ROC analysis; Youden index; lipid thresholds. |
| **Methods: Statistical methods** | 12a | Describe all statistical methods, including those used to control for confounding. | 11–12 | Wilcoxon Kruskal-Wallis test, logistic regression, FDR correction, AIC, Spearman correlation, ROC analysis, and exercise adjustment. |
|  | 12b | Describe methods used to examine subgroups and interactions. | 11–16 | Analyses stratified by sex, SLD status, and obesity status; exercise-adjusted models. |
|  | 12c | Explain how missing data were addressed. | 11 | No missing data for variables included in the analyses. |
|  | 12d | Describe analytical methods accounting for sampling strategy, if applicable. | NA | Not applicable. |
|  | 12e | Describe any sensitivity analyses. | 13–16 | Obesity-stratified analyses, exercise-adjusted analyses, AUS score analyses, and evaluation in an independent cohort. |
| **Results: Participants** | 13a | Report numbers of individuals at each stage of the study. | 6–7, 12, 36, 48 | 312 screened; 59 excluded; 253 included. Figure 1. |
|  | 13b | Give reasons for non-participation at each stage. | 6–7, 36, 48 | Eligibility criteria and Figure 1 report lack of consent and clinical exclusion criteria. |
|  | 13c | Consider use of a flow diagram. | 36, 48 | Figure 1. |
| **Results: Descriptive data** | 14a | Give participant characteristics and information on exposures and potential confounders. | 12–16, 37–44 | Participant characteristics and Tables 1–4. |
|  | 14b | Indicate number of participants with missing data for each variable. | 11 | No missing data for variables included in the analyses. |
|  | 14c | Summarize follow-up time for cohort studies. | NA | Not applicable to this cross-sectional study. |
| **Results: Outcome data** | 15 | Report numbers of outcome events or summary measures for a cross-sectional study. | 12–16, 37–47 | SLD prevalence, lipid measures, genotype distributions, and Tables 1–6. |
| **Results: Main results** | 16a | Give unadjusted and adjusted estimates with precision; state confounders. | 14–15, 45–46 | Table 5 and accompanying Results; exercise-adjusted analyses in Table S6. |
|  | 16b | Report category boundaries when continuous variables were categorized. | 7, 9–10, 15–16, 47 | BMI ≥25 kg/m²; AUS score ≥2; HDL-C <64 mg/dL; LDL-C ≥114 mg/dL. |
|  | 16c | Translate relative risk into absolute risk when relevant. | NA | Not applicable. |
| **Results: Other analyses** | 17 | Report subgroup, interaction, and sensitivity analyses. | 13–16, 39–49 | SLD- and obesity-stratified analyses; exercise-adjusted models; AUS score analyses; independent cohort evaluation. |
| **Discussion: Key results** | 18 | Summarize key results with reference to study objectives. | 17 | Opening paragraph of Discussion. |
| **Discussion: Limitations** | 19 | Discuss limitations, sources of bias or imprecision, and direction and magnitude where relevant. | 22–24 | Single-country setting, selection/generalizability, low female SLD prevalence, residual confounding, exercise questionnaire, and absence of quantitative liver-fat measurement. |
| **Discussion: Interpretation** | 20 | Give a cautious overall interpretation considering objectives, limitations, multiplicity, and other evidence. | 17–24 | Discussion and Conclusions distinguish SLD prevalence from genotype-lipid associations and emphasize exploratory interpretation. |
| **Discussion: Generalisability** | 21 | Discuss generalisability or external validity. | 22–24 | Limitations discuss the Japanese university health-checkup population and limited representation of the general adolescent population. |
| **Other information: Funding** | 22 | Give funding sources and the role of funders. | 26–27 | Funding declaration lists MHLW and AMED support and states that funders had no role in study conduct or reporting. |

NA, not applicable; AUS, abdominal ultrasonography; SLD, steatotic liver disease; FDR, false discovery rate; AIC, Akaike information criterion.

*Give information separately for cases and controls in case-control studies and, if applicable, for exposed and unexposed groups in cohort and cross-sectional studies.

**Note:** An Explanation and Elaboration article discusses each checklist item and gives methodological background and published examples of transparent reporting. The STROBE checklist is best used in conjunction with this article (freely available on the Web sites of PLoS Medicine at http://www.plosmedicine.org/, Annals of Internal Medicine at http://www.annals.org/, and Epidemiology at http://www.epidem.com/). Information on the STROBE Initiative is available at www.strobe-statement.org.
